# Plants used traditionally to treat malaria in Brazil: the archives of Flora Medicinal

**DOI:** 10.1186/1746-4269-3-18

**Published:** 2007-05-01

**Authors:** Alexandros S Botsaris

**Affiliations:** 1 IBPM (Brazilian Institute of Medicinal Plants), Rua Gal Urquiza 128, Rio de Janeiro, RJ, 22431-040, Brazil

## Abstract

The archives of Flora Medicinal, an ancient pharmaceutical laboratory that supported ethnomedical research in Brazil for more than 30 years, were searched for plants with antimalarial use. Forty plant species indicated to treat malaria were described by Dr. J. Monteiro da Silva (Flora Medicinal leader) and his co-workers. Eight species, *Bathysa cuspidata*, *Cosmos sulphureus*, *Cecropia hololeuca*, *Erisma calcaratum*, *Gomphrena arborescens*, *Musa paradisiaca*, *Ocotea odorifera*, and *Pradosia lactescens*, are related as antimalarial for the first time in ethnobotanical studies. Some species, including *Mikania glomerata*, *Melampodium divaricatum*, *Galipea multiflora*, *Aspidosperma polyneuron*, and *Coutarea hexandra*, were reported to have activity in malaria patients under clinical observation. In the information obtained, also, there were many details about the appropriate indication of each plant. For example, some plants are indicated to increase others' potency. There are also plants that are traditionally employed for specific symptoms or conditions that often accompany malaria, such as weakness, renal failure or cerebral malaria. Many plants that have been considered to lack activity against malaria due to absence of in vitro activity against *Plasmodium *can have other mechanisms of action. Thus researchers should observe ethnomedical information before deciding which kind of screening should be used in the search of antimalarial drugs.

## 1. Background

Flora Medicinal is an ancient and small pharmaceutical laboratory established, in early 1915, by Mr. José Monteiro da Silva, a Medical Doctor in Rio de Janeiro. Mr. Monteiro da Silva was an idealist who believed that the Brazilian rainforest had an enormous potential for research and discovery of new drugs. For more than 40 years Mr. Monteiro da Silva had organized a group of technicians and scientists who made a great number of excursions into Brazilian rainforest, collecting plant specimens and information. Although he had also edited the Revista da Flora Medicinal, a scientific paper in which he described his discoveries, a considerable part of his research remains unpublished. During the '30 s and '40 s, the Revista da Flora Medicinal was translated to French and republished by the Institut Pasteur, in Paris, which allowed some of his findings to be used by the international pharmaceutical industry. During his activities, Mr. Monteiro da Silva and his team described more than 200 new medicinal plants from this region. One of his targets was the study of new antimalarial plants, as at his time malaria was a concerning health problem in Brazil. In the following years, quinine, its derivatives and other drugs helped to control malaria. Nowadays, however, its incidence is again growing worldwide, and *Plasmodium falciparum *is getting more resistant to the usual antimalarial drugs[1]. It is estimated that 62% of *P. falciparum *around the world presents with mono or multiresistant drug profile[1]. The World Health Organization estimates that there are between 300 and 500 million new cases of malaria worldwide, every year, mostly in Africa, Asia, South Pacific Islands and South America, which causes, at least, 3 million deaths[2,3]. The main drugs developed for malaria and used up to now (quina alkaloids derived drugs and artemisinin) were discovered based on traditional use and ethnomedical data[4,5]. New efforts to search for novel drugs for treating malaria are very important in countries like Brazil, where many endemic areas still exist[6]. The study of well-documented data such as the archives of Flora Medicinal can point out traditional and probably effective treatments that had not been yet subjected to testing.

## 2. Materials and methods

All documents, including books, hand notes, unpublished studies and the issues of Revista da Flora Medicinal, belonging to the library of Mr. Monteiro da Silva, were examined for information about botanical therapies and plant species used for malaria. Any data or references to plants used for malaria were carefully inserted into a template, and botanical name and classification were re-examined and confirmed with four major plant databases – The Missouri Botanical Garden's VAST[7], the International Plant Names Index[8], the New York Botanical Garden vascular plants database[9] and the Brazilian's Northeast Plants Database[10]. Other information existing in modern databases such as Pubmed (U.S. National Library of Medicine's database that is searchable on the Web) were also examined and compared to other ethnopharmacological studies and current published data.

A review of plants with possible antimalarial activity reported in ethnomedical studies or in pharmacological and biochemical research was also made [11-31].

## 3. Results

The results are summarized on Table 1. Forty [40] plants with possible antimalarial activity were reported and examined by Dr. Monteiro da Silva and his co-workers. The plants were identified by scientific names and families, as well as by vernacular names and usual translations to English, if existent. For each of the species, the parts used for general conditions and symptoms and for treating malaria, as gathered from ethnomedical reports published in Flora Medicinal, are listed. Scientific data about in vitro and in vivo research are also provided.

**Table 1:** Plants with possible antimalarial activity gathered from ethnomedical reports published in Flora Medicinal

**Scientific name and family**	**Vernacular name**	**Part used**	**General indications found in ethnomedical studies**	**Information regarding use for malaria**	**Scientific data on anti-malarial activity**	**Origin and geographic distribution**	**Ref.**
*Aniba canelilla *(H.B.K.) Mez. (Lauraceae)	Preciosa, Casca preciosa, Pau rosa, Casca do Maranhão, Rosewood, Brazilian rosewood	Barks and leaves	Arthritis, fever, colic, heart problems, dyspepsia, infection, intermittent fevers, weakness, malaria, leukorrhea, chronic discharge. Thoracic, stimulant.	Dr. Monteiro da Silva indicates that Amazon Indians used this plant to treat malaria.		Large tree that occurs in the Amazon and Atlantic Forest.	(32)
*Acanthospermum australe *(Loefl.) Kunt. (Asteraceae)	Amor de negro, Mata-pasto, Picão da praia, Picão da prata, Paraguayan starburr	Leaves and roots	Fever, malaria, diarrhea, erysipelas, anemia, urinary infections, blennorrhea, bronchitis, dyspepsia. Tonic, diaphoretic, eupeptic, antidiarrheal, mucilaginous, antimalarial, antiblennorrhagic.	Dr. Monteiro da Silva indicates this plant as a substitute for quina and reports that doctors have a good outcome when using this species in malaria.	Plants of the same genus used to treat malaria in Africa showed antiplasmodial activity against *P. falciparum *in vitro.	Herbaceous, invasive and ruderal plant that usually invades crops and occurs spontaneously in the Cerrado.	(33,34)
*Aristolochia cymbifera *Mart. & Zucc. (Aristolochiaceae)	Jarrinha, Mil homens	Roots	Asthma, fever, diarrhea, dyspepsia, gout, infection, amenorrhea, orchitis, intermittent fevers.	Dr. Monteiro da Silva reports that this must be associated to *Cayaponia tayuya *for use in malaria.			(35,36)
*Aspidosperma polyneuron *Müll. Arg. (Apocynaceae)	Peroba rosa, Sobro, Peroba amargosa	Barks	Fever, diarrhea. Febrifuge, antimalarial, astringent.	Indicated for malaria in the Flora Medicinal literature. Plant contains alkaloids with antimalarial action. Cases of malaria controlled with this bark are reported.		Its alkaloids were extensively studied.	(37)
*Bathysa cuspidata *(St. Hil.) Hook. f. (Rubiaceae)	Quina do mato	Barks	Febrifuge, bitter tonic, eupeptic used as a substitute for quina in malaria	Indicated for malaria in the Flora Medicinal literature.		Tree that occurs in the Atlantic Rainforest.	(38)
*Bidens pilosa *L. (Asteraceae)	Picão, Picão preto, Erva picão, Cuambu, Farmer's Friend, Cobbler's pegs, Beggar's ticks, Pitchforks, Hairy beggarticks	Leaves	Jaundice, fever, hepatitis, leukorrhea, diarrhea, pharyngitis, worms, cough, pneumonia, hepatomegaly. Mucilaginous.	Indicated in many medical texts in the past for malaria. Dr. Monteiro da Silva used for patients that did not respond to quinine.	Preclinical tests revealed strong antiplasmodial activity.	Plant with worldwide distribution.	(33,39)
*Cosmos sulphureus *Cav. Syn. *Bidens sulphurea *(Cav.) Sch. Bip. (Asteraceae)	Picão de flor grande, Picão grande, Beijo de moça, Cosmo amarelo, Yellow cosmos, Klondike Cosmos, Sulphur cosmos, Orange cosmos	Fruits and aerial parts	Jaundice, intermittent fever, splenomegaly. Tonic, hepatic, hepatoprotective.	Indicated for malaria in the Flora Medicinal literature.		Plant bred with ornamental purposes.	(33)
*Cassia fistula *L. (Leguminosae Caesalpinioideae)	Chuva de ouro, Cássia amarela, Cássia imperial, Canafístula, Golden shower, Indian laburnum, Purging fistula, Drumstick tree	Barks, leaves and seeds	Poisons, erysipelas. Febrifuge, purgative, emmenagogue, diuretic, hepatic, skin problems.	Indicated by Dr. Monteiro da Silva as an adjuvant for the treatment of malaria.		Ornamental plant found all over Brazil.	(32)
*Catharanthus roseus *(L.) G. Don. (Apocynaceae)	Vinca, Boa noite, Lavadeira, Vinca rósea, Cape periwinkle, Catharanthus, Church flower, Madagascar periwinkle, Red periwinkle, Rosy periwinkle	Aerial parts	Diabetes, urinary infection, malaria, intermittent fever. Sudorific, diuretic, hypoglycemic, febrifuge.	Indicated as a substitute for quina by Dr. Monteiro da Silva.		Ornamental plant used by the pharmacy industry for obtaining alkaloids.	(33)
*Cecropia hololeuca *Miq. (Cecropiaceae)	Emabaúba, Imbaúba, Embaúba branca, Embaúba prateada, Trumpet tree, Silver embauva, Black embauva, White embauva	Leaves, fruit and sprouts juice	Diuretic, antihypertensive, sedative, refreshing, antiinflammatory, thoracic, healing, expectorant antiasthmatic, cough suppressant, resolutive, antithermal.	Indicated by Dr. Monteiro da Silva as an adjuvant in malaria with very high fever or neurological symptoms.		Tree that occurs in the Atlantic Rainforest.	(40)
*Cedrela fissilis *Vell. (Meliaceae)	Cedro rosa, Cedro vermelho, South American cedar	Barks	Swamp fever, urinary infection, diarrhea. Aromatic, astringent, diuretic, depurative, febrifuge.	Indicated for malaria in the Flora Medicinal literature.		Plant that occurs in the Atlantic Forest.	(41)
*Chondodendron platyphyllum *(St. Hill.) Miers. (Menispermaceae)	Abútua, Bútua, Uva do mato	Roots, barks and leaves	Gases, colic, diarrhea, abdominal pain, verminosis, fever, emesis, nausea, infection, bronchitis, amenorrhea, intermittent fever. Antiasthmatic, bitter tonic, eupeptic.	Use by Indians from the Tupi-Guarani tribe for treating malaria, reported by Dr. Monteiro da Silva.		Isoquinolinic alkaloid-rich plant with antiparasitary activity, natural from the Atlantic Forest.	(42,43)
*Cinchona calisaya *Wedd. (Rubiaceae)	Quina peruana, Casca dos jesuítas, Quina verdadeira, Ledger quinine, Calisaya, Jesuit's powder, Yellow cinchona	Barks	Fever, malaria, eczema. Hair tonic.	Used as the main source of quinin by Dr. Monteiro da Silva. It has quinolinic and quinin derivatives in its composition.		Originary from the Amazon.	(38,44)
*Coffea arabica *L. (Rubiaceae)	Café, Cafeeiro, Coffee, Arabica coffee, Arabian coffee, Abyssinian coffee, Brazilian coffee	Leaves and seeds	Colds, intermittent fever. Clears the blood, diuretic, stimulant, antiasthmatic, digestive, hypoglycemic.	Dr. Monteiro da Silva used the leaves decoction to potentiate other plants with anti-malarial activity.		Plant with African origin, adapted to Brazil.	(45)
*Coutarea hexandra *(Jacq.) Schum. (Rubiaceae)	Quina-quina, Quina-brava, Quina-de-pernambuco, Quineira, Murta do mato	Barks	Intermittent fever, gallbladder stones or problems, digestive problems, colic. Antithermal, antimalarial.	Dr. Monteiro da Silva relates this plant as one of the substitutes for quina and reports cases of malaria cure with its use, some described in the book *Botânica Médica Cearence*, from Dr. Francisco Dias da Rocha.		Plant from the Cerrado used for ornamental purposes.	(33)
*Cuphea ingrata *Hoehne (Lythraceae)	Sete sangrias, Perna de saracura, Mata cana, Pega pinto	Aerial parts and whole plant	High blood pressure, syphilis, dermatoses, intermittent fever, stomachache, rheumatism, venereal diseases, urethral discharge. Depurative, antisyphilitic, cholesterol-reducing, antihemorrhagic, mucous membrane protector, tonic, analgesic.	Indicated for malaria in the Flora Medicinal literature. According to Dr. Monteiro da Silva co-workers, this plant potentiates other antimalarial extracts and help preventing renal and cerebral complications in severe cases.		Herbaceous and ruderal plant that occurs in almost all regions of Brazil, used also for ornamental purposes. It is described in all South and Central America.	Personal writings and archives of Mr. Monteiro da Silva.
*Dipteryx odorata *(Aublet) Willd. (Fabaceae)	Fava de Tonka, Faveira de cheiro, Imburana de cheiro, Cumaru de cheiro, Cumaru de folha grande, Tonka bean, Cumaru, Coumarou, Tonquin bean	Seeds	Antispasmodic, emmenagogue, analgesic, febrifuge, brain stimulating.	In a review, Dr. Monteiro da Silva points this species as having potential use in malaria based on ethnopharmacological reports obtained in his expeditions.		Plant from the Amazon that is rich in coumarins, which gives it a special odor, and for this reason it has been used in the food and tobacco industry as an odorizing agent.	(32)
*Elephantopus mollis *Kunth. (Asteraceae)	Erva grossa, Língua de vaca, Pé de elefante, Elephantopus, Elephant's foot, False tobacco, Tobacco weed	Aerial parts	Fever, jaundice, gallstone, diarrhea, herpes, syphilis, colds, flu, rheumatism, general pruritus. Tonic, depurative.	Indicated for malaria in the Flora Medicinal literature. Some doctors suggest that this plant could be tried if no chinchona bark or substitute is available.		Herbaceous and ruderal plant that is found all over Latin America.	(41)
*Erisma calcaratum *(Link) Warm. (Vochysiaceae)	Jaboti, Erva de Jaboti, Jabuti, Jabuti-araconha, Jabuti da várzea, Jaboty, Jaboty palm	Fruits	Skin infections, dermatoses, fever, malaria. Oleaginous, resolutive.	Reported in review as a plant used by the Amazonian Indians for the treatment of malaria.		Medium sized tree that grows along the moist lowlands of the Amazon.	(32)
*Esenbeckia febrifuga *(St. Hil.) A. Juss. ex Mart. (Rutaceae)	Quina do mato, Angustura, Gumarim	Barks	Malaria, intermittent fever, adenitis, constipation, dyspepsia. Bitter tonic, febrifuge.	Indicated for malaria in the Flora Medicinal literature.		Tree natural from the Southern and Southeast regions.	(38)
*Galipea multiflora *Schultz (Rutaceae)	Quina falsa, Jasmim do mato, Ticoró, Guamixinga	Barks	Dyspepsias, gastric atony, fever, infections, malaria. Tonic, astrigent, bitter, eupeptic, febrifuge, antidiarrheal.	Reported as a substitute for quina in the treatment of malaria in a review by Dr. Monteiro da Silva. Effective in malaria, but weaker than Peruvian chinchona.		Tree that occurs in the Cerrado and Atlantic Forest.	(32,38)
*Geissospermum sericeum *(Sagot) Benth (Apocynaceae)	Pau pereira, Quinarana, Pau forquilha, Acariranha	Barks	Dermatoses, inflammations, swamp fevers. Bitter tonic.	Reported in a review as being a plant tested and approved by doctors for malaria.	Alkaloids with activity against *Plasmodium falciparum *were isolated from trees of the genus *Geissospermum*.	Species from the Atlantic Forest.	(32,42,46)
*Gomphrena arborescens *L. (Amaranthaceae)	Paratudo, Paratudinho, Perpétua raiz do padre	Leaves, flowers and tuberous roots	Weakness, colitis, fevers, mental fatigue, intermittent fevers. Antithermal, antidiarrheal, febrifuge, tonic, emmenagogue, aromatic, eupeptic, antitoxic, protector.	Indicated for malaria in the Flora Medicinal literature. Use in malaria introduced by Brazilian priests that learned it from Indians.		Plant natural from the central region (Cerrado) of Brazil, sometimes cultivated as ornamental.	(47)
*Himatanthus lancifolius *(Mull. Arg) Wood. Syn. *Plumeria lancifolia *Müll. Arg. (Apocynaceae)	Agoniada, Plumeria, Agonium, Arapuê	Barks	Menstrual cramps, fever, hysteria, gastric atony, malaria. Purgative, antispasmodic.	Use for malaria described by Pekolt among Guarani Indians.		Plant that occurs in the Cerrado and Atlantic Forest.	(48)
*Jateorhiza palmata *Miers. (Menispermaceae)	Calumba, Calunga	Barks	Flatulence, colic, diarrhea, abdominal pain, verminosis, fever, emesis, nausea, infection, hypertension, bronchitis, dyspepsia, digestive atony. Bitter tonic, eupeptic.	Plant rich in quinolinic alkaloids with antiparasitary potential.		Exotic plant, natural from Africa, adapted to Brazil.	(41)
*Melampodium divaricatum *(L.C. Rich.) DC. (Asteracea)	Picão da praia, Fel da terra, Salsa da praia, Butter daisy	Leaves	Fever, malaria, flatulence, stomachache, colics, joint pain, muscular pain, palpitation, vertigo, rheumatism, jaundice, anuria. Diuretic, carminative.	Dr. Monteiro da Silva reports many cases of malaria cure using the extract of this plant.		Worldwide distribution.	(32,49)
*Mikania glomerata *Spreng. (Asteracea)	Guaco, Coração de Jesus, Erva de cobra, Cipó almecega	Leaves and flowers	Rheumatism, snake poison, intestinal problems, colics, dysmenorrhea, fever, malaria.	Dr. Pires de Almeida reports to have observed Indians using this plant for malaria with good outcomes.		Liana that is common in the Atlantic Forest.	(50,51)
*Musa paradisiaca *L. (Musacea)	Banana, Bananeira	Stem juice	Worms, diarrhea, intermittent fever, weakness. Tonic, antidiarrheal, thoracic, expectorant, nutritive.	Indicated by Dr. Monteiro da Silva to potentiate other plants used in malaria and help in the recovery of patients.		Exotic plant adapted to Brazil.	(52)
*Ocotea odorifera *(Vell.) Rohwer Syn.*Ocotea pretiosa *(Nees) Mez (Lauraceae)	Sassafraz, Canela de sassafraz, Sassafraz do Brasil, Brazilian sassafras	Barks and roots	Dermatoses, joint pain, fever, rheumatism, syphilis, gout. Sudorific, depurative.	Indicated for malaria in the Flora Medicinal literature. One of the plants used by Guarany Indians to treat fever and malaria.		Species form the Atlantic Forest.	(41)
*Picrolemma sprucei *Hook. f. (Simaroubacea)	Caferana, Caferana verdadeira	Aerial parts and roots	Malaria, intermittent fevers. Sudorific, depurative, febrifuge, antiinfectious.	Dr. Monteiro da Silva reports many cases of recovery from malaria after treatment with the extract of this plant.		Shrub that grows on solid ground in the Amazon.	(49)
*Pradosia lactescens *(Vell.) Radlk. (Sapotaceae)	Bunhanhém, Pau de remo, Pau doce, Guaranhém, Monesia	Barks	Discharge, bronchitis, hemoptysis, diarrhea, ocular inflammation, tuberculosis, cutaneous ulcers, metrorrhagia. Bark provides a milky juice that is astringent and tonic.	Indicated for malaria in the Flora Medicinal literature. According to Dr. Monteiro da Silva, this plant could be associated to any antimalarial therapeutic drug if the patient is not recovering quickly.		Species from the Atlantic Forest.	Personal writings and archives of Mr. Monteiro da Silva.
*Quassia amara *L. (Simaroubacea)	Quassia, Casca amargosa, Pau amargo, Pau de surinã, Quassia-wood, Surinam quassia, Bitter quassia, Bitterwood	Barks	Gastric debility, dyspepsia, blennorrhea, flatulence, fever, malaria, diarrhea, worms. Bitter tonic.	According to a survey by Dr. Monteiro da Silva, it is used by Indians from the North of Brazil and from Suriname for treating malaria.	Extracts showed antimalarial activity in experimental malaria in mice.	Plant from the Amazonian Rainforest.	(53,54)
*Remijia ferruginea *A. St. Hil. (Rubiaceae)	Quina mineira	Barks	Intermittent fever, malaria.	Cited by Dr. Monteiro da Silva as one of the species popularly used to substitute quina in the treatment of malaria.	Tested in mice with experimental malaria caused by *P. berghei*, with reduction of 98% of infected red blood cells.	Medium sized tree that occurs in Atlantic Rainforest and South Amazonia.	(33,55)
*Simaba ferruginea *A. St. Hil. (Simarubacea)	Calunga	Barks and roots	Malaria, fevers, diarrhea. Tonic, eupeptic, febrifuge, antidiarrheal, diuretic.	Indicated for malaria in the Flora Medicinal literature. According to Peckolt, it was used by Amazonian Indians to treat malaria.	A quassinoid isolated from *Simaba *sp showed activity against *Plasmodium falciparum *in vitro.	Huge tree from the Amazon Forest.	(41,56)
*Simarouba amara *L. (Simaroubacea)	Calunga, Marubá, Marupá, Dysentery bark, Bitterwood, Slave wood, Bitter damson	Barks and roots	Intestinal infections, verminosis, fever, wounds, infected ulcers, abdominal pain. Antidiarrheal, antispasmodic, healing.	Used by Amazonian Indians to treat fever and malaria. According to Dr. Monteiro da Silva, it can be used in cases with neurological signs.		Big size tree that occurs in the Atlantic Rainforest and South Amazonia.	Personal writings and archives of Mr. Monteiro da Silva.
*Strychnos pseudoquina *A. St. Hil. (Loganiaceae)	Quina do campo, Quina branca, Quineira, Quina-grossa, Quina do cerrado	Barks	Splenomegaly, hepatomegaly, intermittent fever, malaria, gastric problems. Tonic, bitter, febrifuge, depurative.	Cited by Dr. Monteiro da Silva as one of the species popularly used to substitute quina in the treatment of malaria. According to Andrade-Neto, its potency is inferior to Peruvian quina bark and must be associated with other antimalarial plants.	In a test with experimental malaria in chicken caused by *P. berghei*, no activity was found.	Shrub from the Cerrado that produces edible fruits.	(33,38,55)
*Tabebuia avellanedae *Lor. Ex Griseb. (Bignoniaceae)	Ipê roxo, Pau d'arco, Trumpet tree	Barks	Fever, tumors, allergy, weakness, psoriasis. Antiinfectious, antifungic, anticancer, tonic, immunestimulant.	Indicated for malaria in the Flora Medicinal literature. Should be added to antimalarial regimens for weak patients or in cases of renal failure.		Tree that occurs in the Cerrado and Atlantic Rainforest, with strong medicinal uses.	Personal writings and archives of Mr. Monteiro da Silva.
*Tachia guianensis *Aubl. (Gentianacea)	Jacaruaru, Quassia do Pará, Caferana, Tinguá-aba	Branches and roots	Infections, abdominal pain, worms, malaria. Digestive, antiinflamatory, febrifuge.	One of the plants cited by Dr. Monteiro da Silva as having potential for treating malaria. Plant used in Amazon by Indians to treat malaria.			Personal writings and archives of Mr. Monteiro da Silva. (47)
*Tabebuia impetiginosa *Syn. *Tabebuia roseo-alba *(Bignoniaceae)	Ipê preto, Ipê roxo, Ipê rosa, Trumpet tree	Barks	Fever, tumors, malaria, parasitosis. Antiinfectious, antifungic, anticancer, tonic, immunestimulant.	Indicated for malaria in the Flora Medicinal literature. Should be added to antimalarial regimens for weak patients or in cases of renal failure.		Tree that occurs in the Cerrado and Atlantic Rainforest, with strong medicinal uses.	Personal writings and archives of Mr. Monteiro da Silva.
*Xylopia brasiliensis *Spreng. (Annonaceae)	Embira de caçador, Pindaíba	Seeds and barks	Stomachaches, flatulence, malaria. Stomachic, carminative, febrifuge.	Indicated for malaria in the Flora Medicinal literature, but Dr. Monteiro da Silva considered it a weak antimalarial drug.	*Xylopia sp *extracts proved active against *P. falciparum *with IC_50 _between 3 and 10 mcg/ml.	Plant that occurs in the Cerrado and Atlantic Forest.	Personal writings and archives of Mr. Monteiro da Silva. (57)

Most plants, like *Bidens pilosa*, *Cantharanthus roseus*, *Cassia fistula*, *Cinchona calisaya*, *Cuphea ingrata*, *Geissospermum sericeum*, *Jateorrhiza palmata*, *Quassia amara*, *Simaba ferruginea*, and *Strychnos pseudoquina*, were already reported as antimalarial in previous ethnobotanical studies. Some of these had also their activity against *Plasmodium *tested, as shown on Table 1. Eight species are reported as antimalarial for the fist time: *Bathysa cuspidata*, *Cosmos sulphureus*, *Cecropia hololeuca*, *Erisma calcaratum*, *Gomphrena arborescens*, *Musa paradisiaca*, *Ocotea odorifera*, and *Pradosia lactescens*.

A greater proportion of the plants reported as antimalarial belong to the families Asteraceae (six species), Rubiaceae (five), Apocynaceae (four), and Simaroubaceae (four).

## 4. Discussion

Most research for antimalarial new drugs is only focused on direct activity against *Plasmodium *species. But attention to ethnomedical information gathered by Monteiro da Silva suggests that other effects should be investigated. For example, some plants are referred to enhance the action of other herbs, which can indicate an increase on permeability of the *Plasmodium *membrane to antiparasitic substances, or an inhibition of pump mechanisms of eliminating the drugs[58,59]. Considering that one of the common mechanisms of drugs resistance is the reduction of permeability, the development of drugs that enhance parasite permeability could be of valuable help in the treatment of infectious diseases[60,61]. Other possible mechanism of action is interference with parasite enzymes used for protection against antiparasitic drugs[62].

Some plants with noticeable ethnopharmacological use in malaria showed only weak or even no activity against *Plasmodium *in vitro[55]. For example, *Mikania glomerata*, *Melampodium divaricatum*, *Galipea multiflora*, *Aspidosperma polyneuron*, and *Coutarea hexandra *had their antimalarial activity confirmed by clinical observations of medical doctors (Table 1), an information that yields a high probability of accuracy.

Some authors have underestimated the traditional plants used for malaria based exclusively on low activity against *Plasmodium *in vitro or in animal models[55]. This can be a mistake of strategy or even methodology.

There are many explanations for the absence of in vitro activity of an effective antimalarial drug. As an example, the active principle could be formed by hepatic metabolism, or as a result of transformation by gut bacteria. Other possible mechanisms of action include immunomodulation or interference with the invasion of new red blood cells by parasites, which can be species specific. Therefore studies in human subjects, as well as the observance of ethnomedical detailed data, are urged in order to exclude or confirm the activity of herbs traditionally used to treat malaria.
